# The other person’s smiling amount affects one’s smiling response during face-to-face conversations

**DOI:** 10.3389/fnbeh.2024.1420361

**Published:** 2024-08-09

**Authors:** Yota Obayashi, Shintaro Uehara, Akiko Yuasa, Yohei Otaka

**Affiliations:** ^1^Department of Rehabilitation, Fujita Health University Hospital, Aichi, Japan; ^2^Faculty of Rehabilitation, Fujita Health University School of Health Sciences, Aichi, Japan; ^3^Department of Rehabilitation Medicine, Fujita Health University School of Medicine, Aichi, Japan; ^4^Japan Society for the Promotion of Science, Tokyo, Japan

**Keywords:** facial expression, smile, mimicry, conversation, interaction

## Abstract

**Introduction:**

Smiling during conversation occurs interactively between people and is known to build good interpersonal relationships. However, whether and how much the amount that an individual smiles is influenced by the other person’s smile has remained unclear. This study aimed to quantify the amount of two individuals’ smiles during conversations and investigate the dependency of one’s smile amount (i.e., intensity and frequency) on that of the other.

**Method:**

Forty participants (20 females) engaged in three-minute face-to-face conversations as speakers with a listener (male or female), under three conditions, where the amount of smiling response by listeners was controlled as “less,” “moderate,” and “greater.” The amount of the smiles was quantified based on their facial movements through automated facial expression analysis.

**Results:**

The results showed that the amount of smiling by the speaker changed significantly depending on the listener’s smile amount; when the listeners smiled to a greater extent, the speakers tended to smile more, especially when they were of the same gender (i.e., male–male and female–female pairs). Further analysis revealed that the smiling intensities of the two individuals changed in a temporally synchronized manner.

**Discussion:**

These results provide quantitative evidence for the dependence of one’s smile on the other’s smile, and the differential effect between gender pairs.

## 1 Introduction

Smiling during conversations, especially when responding to the other person’s smile, has been considered a behavior representing liking or rapport with the other person (Chartrand and Bargh, 1999; Lakin and Chartrand, 2003; Hess and Bourgeois, 2010; Hess and Fischer, 2014). This behavior is known to occur interactively between persons; one’s smile affects the other’s, and vice versa, thus contributing to building good interpersonal relationships and consequently, social bonds (Hess and Bourgeois, 2010; Heerey and Crossley, 2013).

This smiling interaction can be treated as the recurrent facial mimicry of smiling, in which similar facial expressions appear in response to the emotional facial expressions of others (Seibt et al., 2015). Smiling interaction is thought to be driven by following the psychosocial features of smiling, as follows: (1) Smiling makes the other person’s emotion more positive (Strathearn et al., 2008; McLellan et al., 2012). (2) Smiling gives a positive impression of oneself to the other, such as improved attractiveness and trustworthiness (Mehu et al., 2008; Schmidt et al., 2012). (3) Smiling works as a backchannel to show understanding, agreement, and empathy to the other person (Haakana, 2010; Niewiadomski et al., 2010). These smiling features motivate the other to build an affiliative social bond, leading them to smile interactively (Chartrand and Bargh, 1999; Lakin and Chartrand, 2003; Hess and Bourgeois, 2010; Hess and Fischer, 2014). In addition, smiling interaction is also thought to be driven by social norms that spontaneously control how, when, and where we should smile (Hinsz and Tomhave, 1991; Rychlowska et al., 2015). Therefore, in recent years, smiling interaction can be considered a potential indicator of cognitive impairment and psychological dysfunction, such as in people with dementia who are known to have difficulty maintaining interpersonal relationships (Cheng, 2017; Chiu et al., 2018; Marshall et al., 2018; Sun et al., 2018; Deardorff and Grossberg, 2019). However, despite extensive discussion and research on the psychological and social aspects of smiling interaction, there is little evidence on the extent to which the amount of one’s smile affects (or is affected by) the other’s smile during face-to-face conversations (Hess and Bourgeois, 2010; Heerey and Crossley, 2013; Riehle et al., 2017).

Although previous studies have investigated how one’s smile would be quantitatively affected by the other’s, many were conducted in special experimental settings and not in a natural conversation situation (Niewiadomski et al., 2010; Mauersberger and Hess, 2019; Fujiwara et al., 2022; Zhao et al., 2023). For instance, some studies assessed the frequency of smiles when a participant talked to an artificial human face displayed on a computer screen (Niewiadomski et al., 2010), the activity of smile-related muscles as a marker reflecting the smile intensity when a participant talked to pre-recorded facial movies (Mauersberger and Hess, 2019), or the smile intensity during conversations on the screen with an interviewer (Fujiwara et al., 2022) or during working on cooperative tasks with the other (Zhao et al., 2023). Moreover, some studies have investigated the smiles of pairs of participants during natural daily face-to-face conversations and demonstrated that the frequency of one’s smile affects the other’s, with their smiles synchronizing with each other (Hess and Bourgeois, 2010; Heerey and Crossley, 2013; Riehle et al., 2017). However, Heerey and Crossley (2013) have demonstrated smile-timing synchronization based on the presence or absence of smiles—meaning that it is based on binary information. Although some studies have shown synchronization based on the intensity of muscle activation using electromyography (EMG) as an alternative smile measure (Hess and Bourgeois, 2010; Riehle et al., 2017), the measurement of facial muscles using EMG has some limitations, such as crosstalk and interference (Hess, 2009). The activity of one of the major smile-related muscles, the zygomaticus major, is weak and thus easily contaminated by the activity of nearby muscles involved in mastication, which are activated during speaking. In addition, electrodes attached to the face may interfere with natural facial movements. In particular, the implementation of EMG is technically demanding and time-consuming for preparation and measurement when considering future applications in clinical settings targeting dementia patients, for example.

To address these gaps, we evaluated the intensity and timing of two individuals’ smiles during face-to-face conversations using automated software for facial expression analysis, which can continuously quantify smiles, thus track time-course changes, and investigate the relationship between their smiles. We asked a participant (speaker) to talk to the other (listener) with a pre-determined theme under three conditions, in which the listener controlled their smile amount with “less,” “moderate,” or “greater” extents during conversations. We assessed and compared the amount that the participants’ smiled during the conditions to understand how much one’s smile would affect the other’s. Similar to the results of previous studies, we hypothesized that the amount of a participant’s smile would be regulated depending on the amount of the other’s smile (Hess and Bourgeois, 2010; Niewiadomski et al., 2010; Riehle et al., 2017; Mauersberger and Hess, 2019; Fujiwara et al., 2022). Further, we also hypothesized that their smiles would occur at similar times. The present study also investigated how the gender of the pairs affects these responses. While previous studies have indicated that women tend to express more smiles during conversations than men (Hecht and LaFrance, 1998; Hess and Bourgeois, 2010), the gender effect on one’s smiling response to the other’s smile remains unclear. Although some previous studies have suggested the existence of the gender effect on the smile-receivers’ subjective evaluation of the smile-senders’ traits (Hess et al., 2000; Mehu et al., 2007), these gender effects may be complexly modulated and inconsistent across cultural backgrounds, including gender roles and stereotypes (Mehu et al., 2008; Chaplin and Aldao, 2013). Therefore, we conducted an exploratory investigation into how the other person’s gender influences one’s interacting smile response.

## 2 Materials and methods

### 2.1 Participants

We recruited 42 volunteers for the study. The initial sample size was estimated at 28, using G*Power 3.1.9.6 with the following settings: analysis of variance (repeated measures within factors) for “statistical test,” 0.25 for “effect size *f*,” 0.05 for “α error probability,” 0.8 for “power (1-β error probability),” 4 for “number of groups,” 3 for “number of measurements,” 0.5 for “correlation among repeated measures,” and 1 for “nonsphericity correction ε.” To account for the possible non-parametric distribution of the data, 15% more participants were added (Lehmann and D’Abrera, 1998). As we planned to conduct an exploratory analysis to find a possible trend of gender effects in smiling interaction, a total of 40 participants (20 females, mean and standard deviation [SD] of age: 25.7 [±3.0] years) were recruited as speakers. In addition, two volunteers (one male and one female, 39 and 36 years old) were enrolled as listeners. This study was approved by the Ethics Review Committee of Fujita Health University (approval no. HM21-279) and conducted in accordance with the principles of the Declaration of Helsinki, as revised in 2013. All experimental procedures were conducted after written informed consent was obtained from all participants.

### 2.2 Experimental setup and protocol

**Figure 1:**
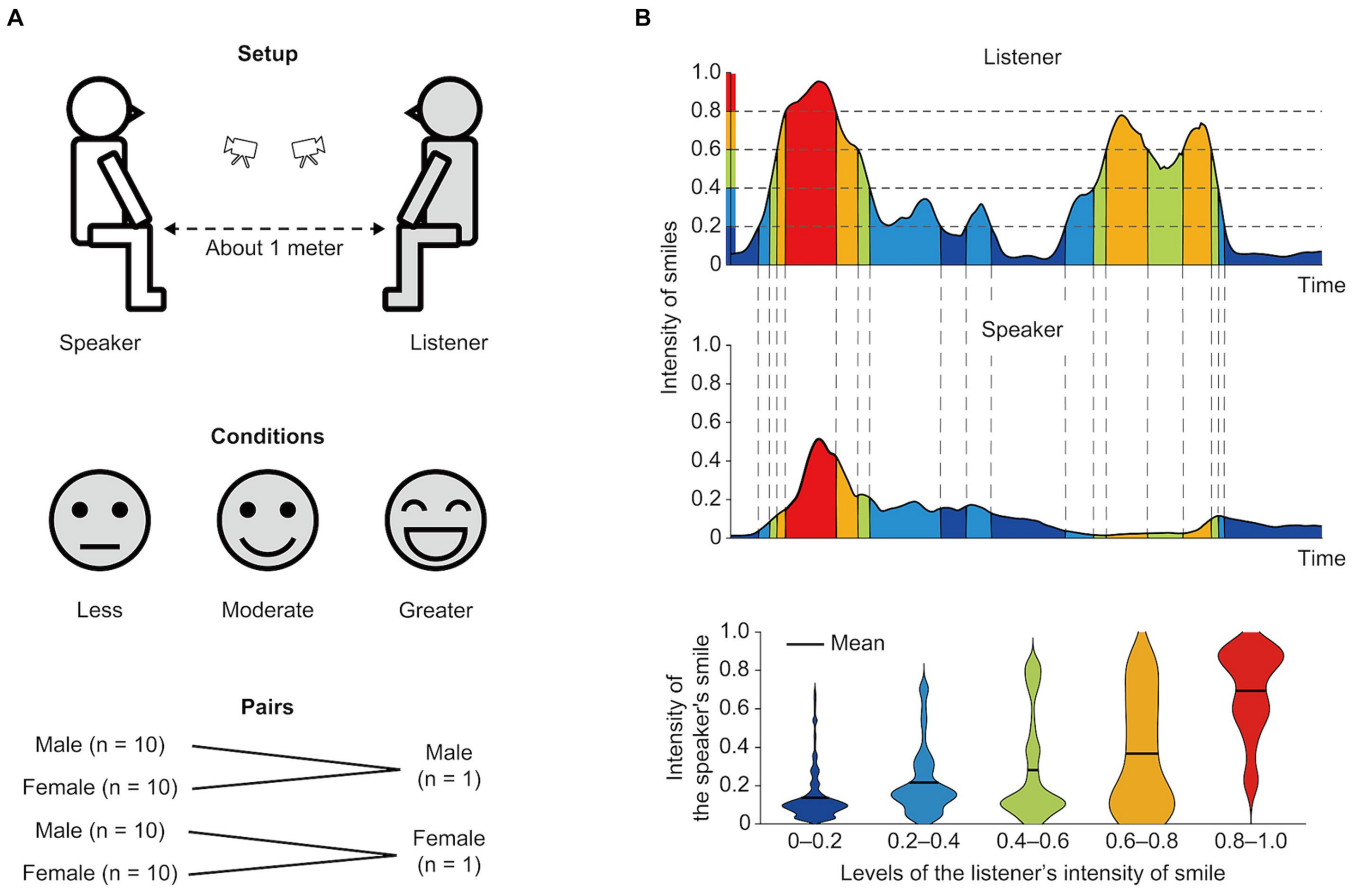
Experimental setups and schematic diagram of data analysis. **(A)** Setup and conditions. A speaker and a listener sat face-to-face at a distance of approximately 1 m apart. Two video cameras were placed in front of each participant to capture their faces during their conversations (top panel). The listener regulated their smile intensity and frequency during the conversations at three different levels: less, moderate, and greater (middle panel). Speaker–listener pairs were divided into four, based on gender pairs (bottom panel). **(B)** Analysis of smile synchrony between a speaker and a listener. The temporal changes of the listener’s smile intensity were divided into five levels, 0–0.2 (deep blue), 0.2–0.4 (light blue), 0.4–0.6 (green), 0.6–0.8 (orange), and 0.8–1.0 (red; top panel). Note that the x-axis is limited to a maximum of 30 s for display purposes. For each three-minute conversation, the mean intensity of the speaker’s smile corresponding to each time window of the listener’s smile was computed (middle and bottom panels). The vertical dashed lines indicate the boundaries of the intensity levels. The bottom panel shows distribution plots displaying the intensity of the speaker’s smile. The horizontal black lines indicate the means across data samples.

The speakers were asked to engage in 3-min conversations with the listeners on pre-determined topics. The listeners were asked to provide responses and ask questions to facilitate the conversations. The speaker and listener sat on chairs facing each other with a distance of approximately 1 m between them. A video camera (FDR-AX45; SONY, Tokyo, Japan) was placed in front of each participant to capture their facial movements. The height of the cameras was set to a level lower than that of the face so that the two individuals could see each other’s faces (top panel, Figure 1A).

In the experiment, three smiling conditions were set, based on the amount of smiling response by listeners, “less,” “moderate,” and “greater” (middle panel, Figure 1A). We defined the “amount of smiles” as the integral of the time-course changes in smile intensity during a conversation, which changes with the frequency of smiles and their intensity. The listeners were asked to voluntarily control the frequency and intensity of their smiles during conversations in each condition. In the “less” and “greater” conditions, the listeners decreased or increased the frequency and intensity of smiles, respectively. In the “moderate” condition, they regulated their frequency and intensity at an intermediate level. The listeners were asked to show their smiles context-dependently at appropriate times during conversations, and to regulate the responsiveness to smiles so that the frequency and intensity of smiles matched each level of the three conditions. Prior to the experiment, we set the practice sessions for the listeners to better control the required smiling behavior. In these sessions, the listeners engaged in conversations with an experimenter for each condition and practiced adjusting the amount of smiles to match each condition. The listeners were also informed about the aim of the experiment. We instructed the speakers and listeners to maintain the position of their faces and bodies as much as possible during conversations, and not to cover their faces with their hands during involuntary actions, such as scratching their cheeks, to enable us to capture clear facial expressions.

Speakers were divided into male and female groups (20 individuals in each). Next, 10 of each group had conversations with the male or female listener. In other words, we prepared four types of gender pairs (speaker–listener): male–male, female–male, male–female, and female–female (bottom panel, Figure 1A). We confirmed in advance that the listeners and speakers have not had previous conversations with each other and that they met for the first time at the time of the experiment. The contents of the conversations were pre-determined, and speakers were asked to talk about the following themes: activities on holidays, their hometowns, and their life histories. These were chosen as topics that were thought not to affect the amount of smiles, *per se*. Each speaker conducted three conversations of 3 min, each of which was conducted in one of the three smile conditions. The order of conditions and conversation topics was randomized across speakers. All speakers were blinded to the experimental conditions throughout the study, but the listeners were informed of the condition before starting a conversation.

We asked speakers to evaluate their feelings with two questionnaires: “How friendly did you feel the listener was to you?” and “How much did you enjoy this conversation?” after each conversation. Speakers rated their experience on an 11-point numerical rating scale (NRS) from 0 (not friendly or enjoyable) to 10 (supremely friendly or enjoyable).

### 2.3 Data analysis

We analyzed the time-course change in the smile intensity during conversations from the captured facial expressions with the automated facial expression analysis software, FaceReader (version 7; Noldus Information Technology, Wageningen, the Netherlands). This software automatically detects changes in facial expressions, based on the facial action coding system (FACS), which is widely used in the field of psychology to analyze facial expressions (Skiendziel et al., 2019). The FACS is an observer-based analysis method that describes visually identifiable facial muscle movements as action units (AUs; Cohn et al., 2007). For example, AU1 represents an inner brow raise and AU9 represents a nose wrinkle. The intensity of an AU is evaluated on a 6-point scale, including not active: the higher the intensity, the more intense the movement of the facial muscles. Based on FACS criteria (Ekman, 1970, 1971; Ekman and Friesen, 1971; Ekman et al., 1987; Ekman and Cordaro, 2011), six emotions (happy, sad, angry, surprised, fearful, and disgusted) related to specific facial expressions have been identified with good validity and reliability (Sayette et al., 2001; Cohn and Ekman, 2005). FaceReader has been reported to be able to detect the emergence of AUs as accurately as certificated FACS coders (Lewinski et al., 2014; Skiendziel et al., 2019) and classify the six emotional facial expressions with high accuracy (Nelson and Russell, 2013; Lewinski et al., 2014; Skiendziel et al., 2019). Therefore, using this software enables non-skilled users to quantitatively evaluate facial expressions and the intensity of related emotions (Obayashi et al., 2021). Note that FaceReader analyzes the facial expressions based on observation and classifies emotion labels, but not the actual emotions of the person being analyzed. In the present study, we focused specifically on the intensity of “happy,” an expression extracted by the software as an indicator of smile intensity. The extracted values ranged from 0 (no smile) to 1 (greatest smile) mainly depending on the levels of AU6 (cheek raise) and AU12 (lip corner pull). The present study did not directly analyze the activity of AUs as a proxy for smile intensity because a multiple/complex combination of AUs must be distinguished to estimate the smile types contributing to social bonding. For example, while AU6 and AU12 are used to estimate the Duchenne smile, only AU12 is used for the social smile (Hess and Bourgeois, 2010). Similarly, it has been shown that the reward smile can be estimated using AU10, AU12, AU14, and AU25, and the affiliation smile can be estimated using AU10, AU12, and AU14 (Zhao et al., 2023). In addition, the combination of AU4, AU6, and AU12 is used for the calculation of the positive pattern score as an indicator of smiles (Mauersberger et al., 2020). Therefore, the present study adopted “happy,” a positive expression estimated by FaceReader, as a global index representing smile intensity.

Overall, 240 clips were analyzed: we captured the faces of speakers and listeners in a total of 40 pairs with three conditions. To check how many percentages of the video frames of the 3-min conversation were utilized in the following analysis, we calculated, in each video clip (duration: 3 min), the percentage of the frame where the software successfully recognized a human face and therefore was able to estimate the smile intensity. The mean usage percentage of the 240 video clips was 99.9% (±0.2), indicating that most of the facial images were not disturbed by noises that might happen when a participant shifted the facial position or covered the face with his/her hand.

As a proxy for the amount of smiles during 3-min conversations, we calculated the mean of the time-course changes of individual speakers for each condition. To investigate the differences in the amount among the conditions, we compared the medians of the speakers (*n* = 40) between the three conditions using the Friedman test. Furthermore, we performed these comparisons separately for each of the four types of speaker–listener pairs: male–male (*n* = 10), female–male (*n* = 10), male–female (*n* = 10), and female–female (*n* = 10), to investigate the influence of the other’s gender on one’s smiling response in each type of pair. Note that, while a mixed ANOVA with linear contrasts seemed appropriate to examine the interaction effect between the gender composition and smile amount condition, we decided to perform the Friedman for each pair type due to the following two reasons: (1) The values for a statistical comparison (i.e., the mean of the speakers’ smiles) did not follow a normal distribution. (2) The number of participants in each gender pair was 10, which was insufficient to estimate the interaction. Instead, the fact that the number of participants across all groups was the same (all 10) and that the *p*-value depends on the number of data allowed us to treat the *p*-value obtained from the Friedman test as a comparable indicator between groups and to indirectly estimate the interaction effects.

We also calculated the mean intensity of the listener’s smile for each conversation and compared the median of the 40 trials (40 conversations) after pooling the two listeners’ data (20 trials each) between the three conditions, using the Friedman test. This supplementary analysis was performed to confirm that the listeners’ amount of smiles was well-controlled to match the pre-set conditions.

We further investigated whether the intensity of the speaker’s smiles was also temporarily dependent on the listener’s smile intensity (Figure 1B). We first separated the intensity of the listener’s smile into five levels, with values of 0–0.2, 0.2–0.4, 0.4–0.6, 0.6–0.8, and 0.8–1.0. We then calculated the mean intensity of the speaker’s smiles during the period in which the listener’s smile intensity was within each of the five levels. The mean was calculated after pooling all data including all three (“less,” “moderate,” and “greater”) conditions. To investigate the extent to which the speaker’s smile intensity was influenced by the listener’s smile intensity, we computed the Spearman’s correlation coefficient between the five smile intensity levels and the mean intensity of the speaker’s smile in each gender pairs. We then performed a one-sample Wilcoxon signed-rank test on the correlation coefficient (i.e., established whether the correlation coefficient was significantly greater than “0”) to evaluate the presence of significant relationships at a group level.

The NRS scores regarding the speakers’ feelings of friendliness and enjoyment were compared among the three conditions using the Friedman test. All statistical analyses were performed using SPSS version 26 (IBM Corp., Armonk, New, United States). Differences with a *p-*value below 0.05 were considered statistically significant. The data used for the statistical analysis are available in the Supplementary material.

## 3 Results

**Figure 2:**
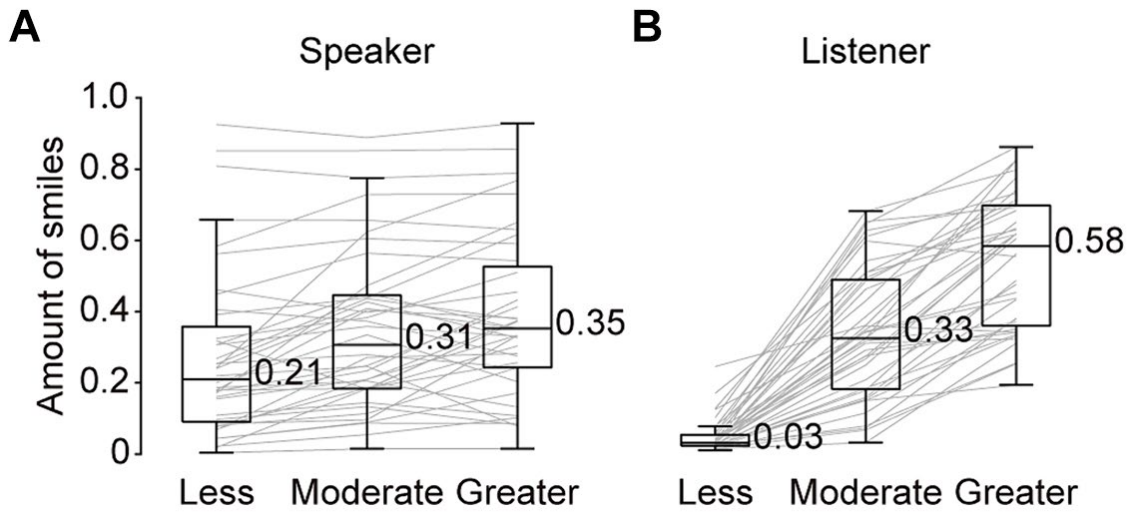
Amount of smiles during the three-minute conversations in each condition for speakers **(A)** and listeners **(B)**. Values near the boxplots indicate the median across speakers **(A)** or trials **(B)**. Gray lines represent individual data.

**Figure 3:**
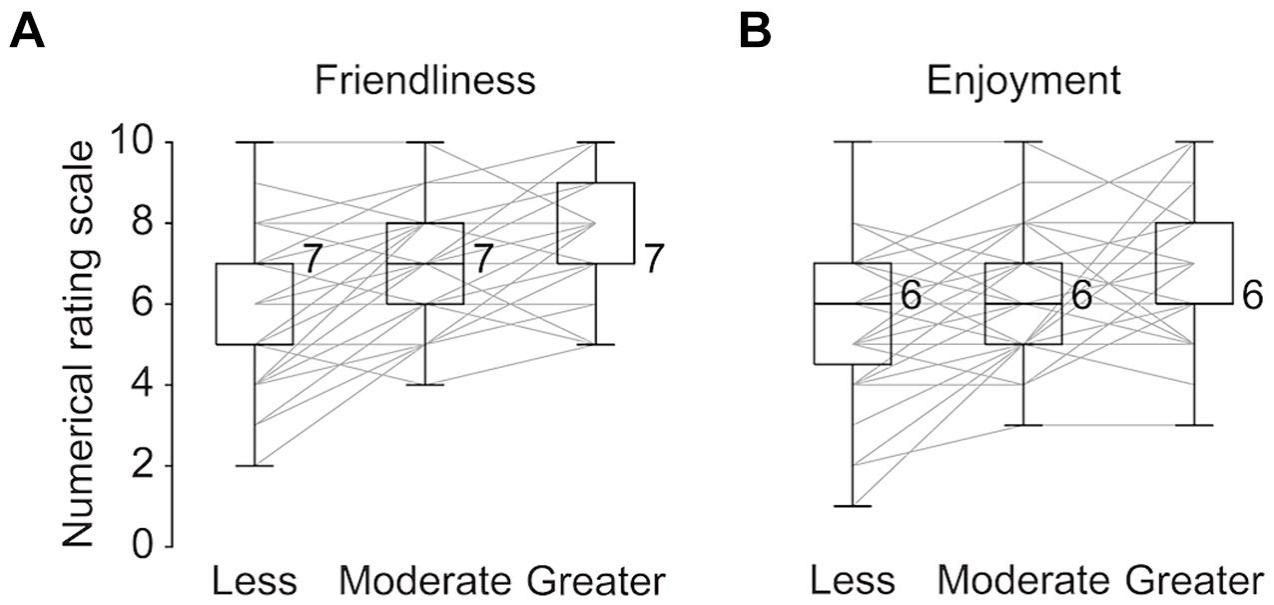
Numerical rating scale for the speaker’s feelings of friendliness **(A)** and enjoyment **(B)** in each condition. Values near the boxplots indicate the median across speakers. Gray lines represent individual data.

We found that the speakers’ smile amount during 3-min conversations were significantly different among the three conditions (χ^2^[2] = 23.55, *p* < 0.001, Kendall’s W = 0.29; Figure 2A): the median amount of smiles showed stepwise increases from the “less” to “greater” conditions. In line with these differences, the amount of the listeners’ smiles was found to be well controlled to match each condition, in that the amount showed gradual increases from the “less” to “greater” conditions (χ^2^[2] = 76.20, *p* < 0.01, Kendall’s W = 0.95; Figure 2B). In addition, we confirmed that speakers’ feelings of friendliness regarding the listeners’ attitudes were regulated depending on the amount of the listeners’ smiles (χ^2^[2] = 24.67, *p* < 0.01, Kendall’s W = 0.31; Figure 3A). Regarding speakers’ feelings of enjoyment of the conversations, we further found a significant difference among the three conditions (χ^2^[2] = 12.79, *p* < 0.01, Kendall’s W = 0.16; Figure 3B). These results demonstrate that one’s smile amount can be influenced by the other’s during natural conversations, with one’s impression of the other person and conversation changing as well.

**Figure 4:**
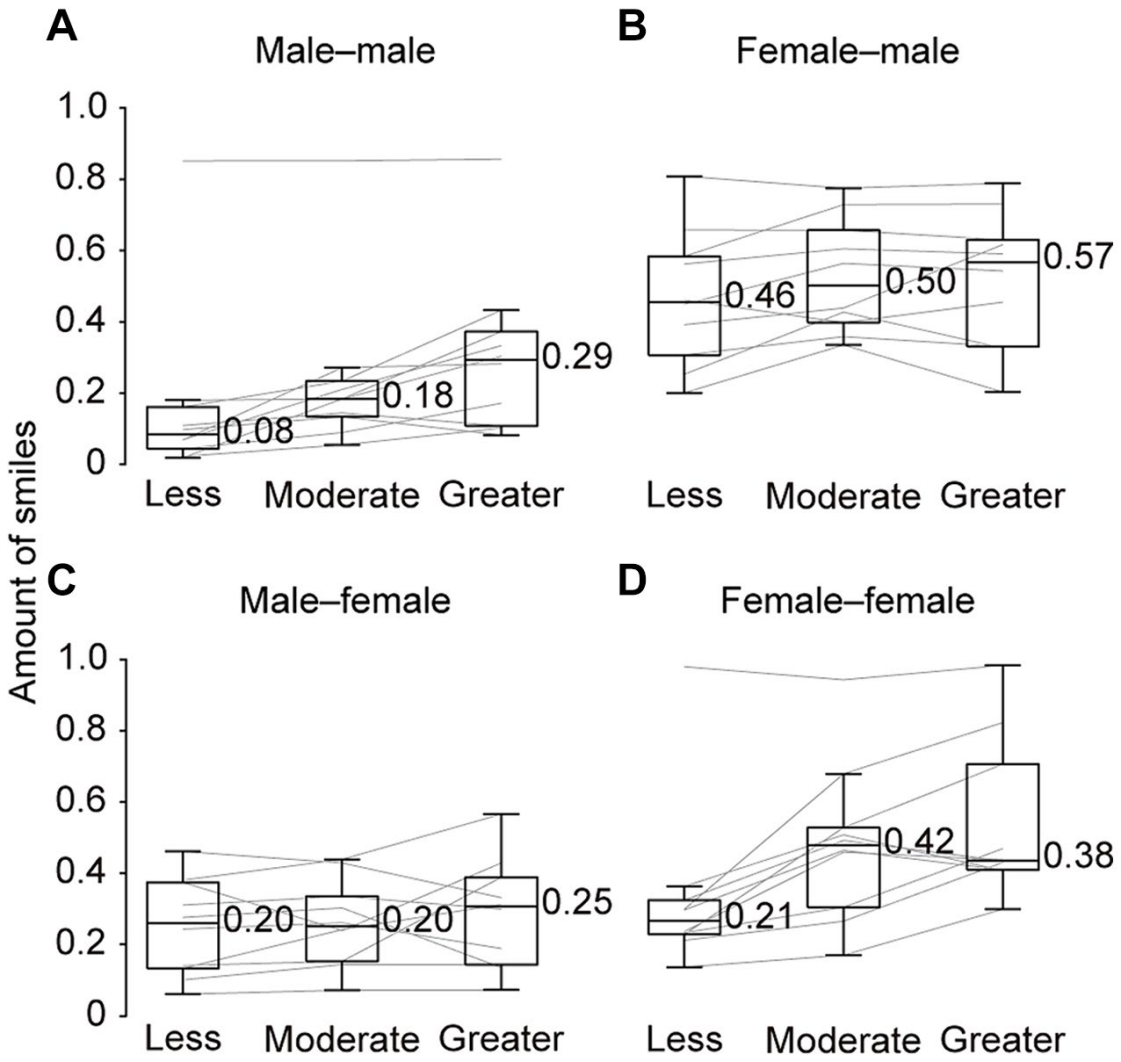
Amount of smiles of speakers during the three-minute conversations in each condition. Values are shown separately for each of the four types of speaker–listener pairs: male–male **(A)**, female–male **(B)**, male–female **(C)**, and female–female **(D)**, respectively. The values near the boxplots indicate the median across speakers. Gray lines represent individual data.

To investigate the differences among gender pairs in smiling responses, we performed the same comparisons separately for each of the four types of speaker–listener pairs. In the same-gender pairs, we found a gradual increase in the amount of smiles from the “less” to “greater” conditions (male–male pair: χ^2^[2] = 10.40, *p* < 0.01, Kendall’s W = 0.52, female–female pair: χ^2^[2] = 12.60, *p* < 0.01, Kendall’s W = 0.63; Figures 4A,D). In contrast, in the different-gender pairs, no clear difference was found among the conditions (female–male pair: χ^2^[2] = 2.60, *p* = 0.27, Kendall’s W = 0.13, male–female pair: χ^2^[2] = 2.60, *p* = 0.27, Kendall’s W = 0.13; Figures 4B,C). This further investigation indicates that the pattern of responses to the others’ smiling may differ, depending on the gender pairs.

**Figure 5:**
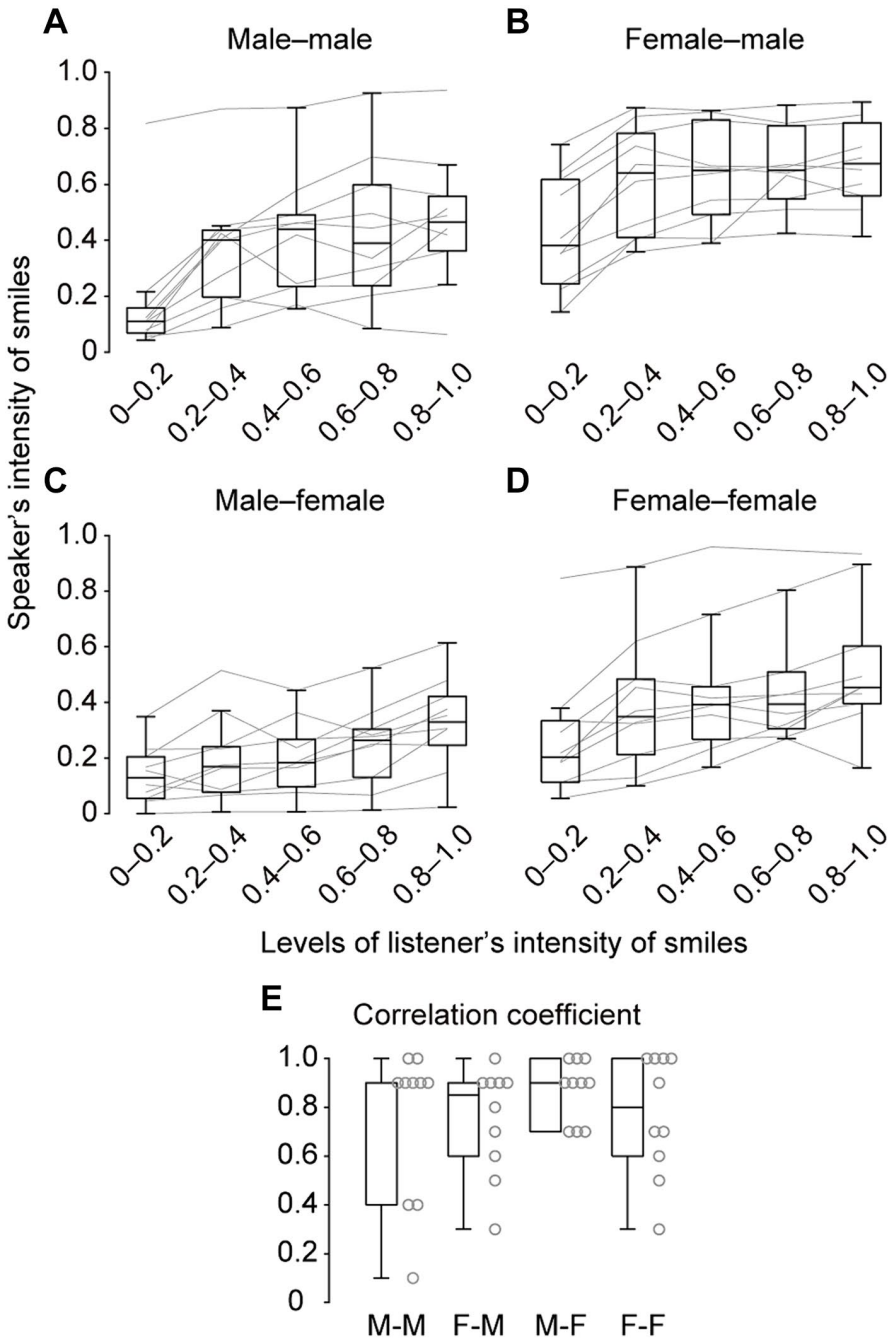
Speakers’ mean smile intensity when the level of a listener’s smile intensity fell within each range: 0–0.2, 0.2–0.4, 0.4–0.6, 0.6–0.8, and 0.8–1.0. The data were shown separately for each of the four types of speaker–listener pairs: male–male **(A)**, female–male **(B)**, male–female **(C)**, and female–female **(D)**, respectively. The gray lines indicate individual data. **(E)** Spearman’s correlation coefficient between the mean intensity of the speaker’s smile and the intensity (five levels) of the listener’s smile for each of the four gender pairs: male–male (M-M), female–male (F-M), male–female (M-F), and female–female (F-F). The gray dots next to the boxplots represent individual data.

Our additional analysis revealed significant positive correlations in each of the four types of speaker–listener pairs (one-sample Wilcoxon signed-rank test: male–male pair: *p* < 0.01, *Z* = 2.84, *r* = 0.90, female–male pair: *p* < 0.01, *Z* = 2.82, *r* = 0.89, male–female pair: *p* < 0.01, *Z* = 2.84, *r* = 0.90, female–female pair: *p* < 0.01, *Z* = 2.82, *r* = 0.89, Figure 5). These results suggest that the presence of smiles and their intensity between speakers and listeners may be temporarily related.

## 4 Discussion

In the present study, we quantitatively analyzed the amount (i.e., integration of smile intensity and frequency) of speakers’ and listeners’ smiles during a conversation, to thereby understand the extent to which the amount of a speaker’s smile is affected by that of the listener’s smile. We found significant differences in the smile amount depending on the listener’s smile amount: when a listener smiles to a greater extent, the speaker tends to smile more, which is specifically found in same-gender pairs.

It is empirically well known in our daily lives that when one person smiles, the other person smiles, and vice versa. However, only a few quantitative studies have measured this smiling interaction in a natural conversation situation (Hess and Bourgeois, 2010; Heerey and Crossley, 2013; Riehle et al., 2017). The present study demonstrates that the speakers’ smile amount during three-minute-long conversations becomes gradually higher from the “less” to the “greater” conditions; namely, the more a listener smiles, the more a speaker smiles. These findings, based on the direct capture of facial expressions, provide quantitative evidence that partly supports the mutual effects of smiling responsiveness during daily face-to-face conversations.

The subjective investigation revealed that speakers’ feelings of friendliness toward the listeners and enjoyment of conversations also increase in conditions where the listeners smile more frequently. These results suggest that speakers’ feelings can be regulated by the listeners’ smile amount, as well as speakers’ outward facial expressions. These results fit well with previous studies, suggesting that the other’s smiles lead to a pleasurable state by activating the reward center in the brain of the person who receives a smile (Strathearn et al., 2008; McLellan et al., 2012). In addition, responding to a smile with a smile is thought to raise affinity and help people build rapport with each other (Chartrand and Bargh, 1999; Lakin and Chartrand, 2003; Hess and Bourgeois, 2010; Hess and Fischer, 2014). These positive psychosocial states would result in a person smiling more (Hinsz and Tomhave, 1991). It is also plausible that the speakers’ self-image is satisfied when a listener smiles more, as the behavior acts as a backchannel to convey agreement or empathy, resulting in positive psychological states (Niewiadomski et al., 2010).

The regulation of the speaker’s smile amount depending on the listener’s smile was evident only in same-gender pairs. In contrast, in the different-gender pairs, speakers tended to express relatively more smiles, even in conditions where listeners smiled less (Figures 4B,C). As partly supporting the present finding, a previous study suggested that the amount of facial mimicry of smiling during conversations was qualified by the gender composition of the pairs and the emotional content of the conversations (Hess and Bourgeois, 2010). Because the content of the conversations was pre-determined, our findings may reflect the regulation of the smile amount derived from the gender difference, independent of the emotional content of the conversation. One of the possible explanations for this regulation could be the lower peer context and stricter social norms that may be present in the different-gender pairs compared with same-gender pairs (Maccoby, 1990; Rose and Rudolph, 2006; Chaplin and Aldao, 2013). We speculate that speakers in the different-gender pairs might be more motivated to express smiles, even when listeners are not smiling, in order to actively build an interpersonal relationship in accordance with social norms (Martin et al., 2017). Another possibility would be that biological motivation influences smiling even in the “less” condition in the different-gender pairs. This view is supported by some previous findings that people are more likely to smile at a person of the opposite sex in the context of a romantic relationship (Mehu et al., 2008; Dosmukhambetova and Manstead, 2012).

Regarding the temporal relationship between speakers’ and listeners’ smile intensities, our additional analysis revealed significant correlations that are similar to those found in previous studies (Hess and Bourgeois, 2010; Heerey and Crossley, 2013; Riehle et al., 2017): both speakers and listeners showed synchronous smiles. Synchronous smiles are thought to occur following psychosocial elements that can be classified into biological (unconscious) and conscious responses. As a biological response, it is thought that a listener’s smile causes a speaker to experience positive emotions, which instantly makes the speaker express more smiles (Strathearn et al., 2008; McLellan et al., 2012; Porter et al., 2012; Hatfield et al., 2014). Regarding conscious responses, there are two perspectives: (1) A person tends to mimic the other’s smiles at the same time to build an affiliative social bond with the other (Chartrand and Bargh, 1999; Lakin and Chartrand, 2003; Hess and Bourgeois, 2010; Hess and Fischer, 2014), regardless of whether they have positive emotions or not (Ekman, 1992; McLellan et al., 2012). (2) People tend to show synchronous smiles because of the social norm in which a smile should be responded to with a smile (Hinsz and Tomhave, 1991; Rychlowska et al., 2015; Wood et al., 2016). In addition, it is plausible that a situation where natural conversations occur makes speakers’ and listeners’ smiles more synchronous (i.e., with less delay in time), given that the smiling response becomes faster when one can predict a conversation’s flow (Heerey and Crossley, 2013). It should be noted that although the listeners were asked to regulate the frequency and intensity of their smiles, they were not asked to regulate their timing. Therefore, the synchronous smiles found in the present study may naturally occur following some psychosocial aspects with less influence from the listeners’ intentions.

The present study used the automated measure for facial expression analysis. This method appears to be simpler than those used in previous studies, such as visual inspection (Heerey and Crossley, 2013) and EMG recordings of facial muscles (Hess and Bourgeois, 2010; Riehle et al., 2017), in that no special equipment, except for a video camera, is needed to identify smiles. In addition, compared to the latter methods, which require special skills, the present automated measure can be performed without special skills. Therefore, our results indicate a first step toward applying the automated facial expression analysis in clinical settings in future studies to measure the smile intensity during a conversation as an indicator of psychological dysfunction or cognitive impairment.

Despite these notable findings, this study has several limitations. This study included one male and one female listener. Therefore, it cannot be denied that listener factors such as appearance, age, and social status may have influenced the speakers’ smile responses (Deutsch, 1990; Adams et al., 2015; Albohn et al., 2019). For example, the fact that both listeners were older than the speakers may have led to a specific social situation that affected the smile responses between them in regions where younger people respect older people, such as East Asia (Tan and Barber, 2020). Furthermore, the speaker factors such as personality, sexual preference, and gender stereotypes were not controlled. Therefore, it is possible that these individual factors biased their smiling (Chaplin and Aldao, 2013; Hess, 2021). Moreover, it should be noted that the number of 40 pairs enrolled in the present study was relatively small, compared to previous studies investigating the smiling mimicry, in which the number of pairs ranged from 30 to 170 (Hess and Bourgeois, 2010; Riehle et al., 2017; Fujiwara et al., 2022; Zhao et al., 2023). In addition, the 40 pairs were then divided into four groups for each gender pair. Therefore, caution should be exercised when interpreting the results, especially those related to the gender difference, due to the limited population. Furthermore, this study could not distinguish between the types of smiles that have a positive or negative influence on building mutual social bonds. A dominant smile type, which displays unilateral asymmetrical activation of the zygomaticus major, has been suggested to have a negative influence on it (Martin et al., 2017). Therefore, smiling responses detected during natural conversations cannot always be treated as positive. In addition, the present study did not classify smile types based on the semantic characteristics between biological smiles with positive emotions and conscious backchannel smiles. Taking these semantic differences among smile types into consideration would deepen the insight into smiling responses to another’s smile, which should be addressed in future studies. Finally, the present study cannot rule out the possibility that eye blinks and facial expressions without smiling worked as a backchannel to convey agreement and empathy and thus facilitated the other to smile more (Seibt et al., 2015; Hömke et al., 2018).

## 5 Conclusion

The present study showed that one’s smile amount during a natural conversation may be significantly affected by the other person’s smile amount in a timely synchronized manner, especially when the conversation is between a same-gender pair. This finding identifies behavioral and psychological aspects that help us understand how smiling contributes to building human social relationships. This finding also suggests a potential maneuver and indicator to quantitatively assess cognitive impairment and psychological dysfunction, such as in people with dementia who have difficulty maintaining interpersonal relationships.

## Data availability statement

The original contributions presented in the study are included in the article/Supplementary material, further inquiries can be directed to the corresponding author.

## Ethics statement

The studies involving humans were approved by the Ethics Review Committee of Fujita Health University. The study was conducted in accordance with the local legislation and institutional requirements. The participants provided their written informed consent to participate in this study.

## Author contributions

YOb: Conceptualization, Data curation, Formal analysis, Funding acquisition, Investigation, Methodology, Resources, Validation, Visualization, Writing – original draft, Writing – review & editing. SU: Conceptualization, Data curation, Formal analysis, Investigation, Methodology, Project administration, Resources, Supervision, Validation, Visualization, Writing – original draft, Writing – review & editing. AY: Investigation, Methodology, Resources, Supervision, Validation, Visualization, Writing – review & editing. YOt: Conceptualization, Methodology, Project administration, Resources, Supervision, Validation, Visualization, Writing – review & editing.
